# Artificial intelligence in trauma care: applications, ethical challenges, and pathways toward responsible integration

**DOI:** 10.1097/ACO.0000000000001615

**Published:** 2026-02-09

**Authors:** Elena Giovanna Bignami, Michele Berdini, Valentina Bellini

**Affiliations:** Anesthesiology, Critical Care and Pain Medicine Division, Department of Medicine and Surgery, University of Parma, Parma, Italy

**Keywords:** artificial intelligence, ethics, governance, machine learning, trauma

## Abstract

**Purpose of review:**

Artificial intelligence is increasingly applied across the trauma care continuum, from prehospital triage to in-hospital decision-making. This review provides a timely synthesis of emerging applications, ethical challenges, and regulatory frameworks shaping the responsible integration of artificial intelligence into trauma systems.

**Recent findings:**

Recent studies highlight the potential of machine learning and deep learning models to improve trauma triage accuracy, imaging interpretation, and prediction of hemorrhage and transfusion needs. Despite promising accuracy, most systems remain in proof-of-concept phases with limited external validation. Ethical and governance challenges – particularly regarding data privacy, transparency, accountability, and automation bias – remain major barriers to clinical translation. The WHO guidance on artificial intelligence ethics and the European Union Artificial Intelligence Act establish core principles of safety, fairness, and human oversight, framing the foundation for trustworthy implementation.

**Summary:**

Artificial intelligence offers transformative opportunities for trauma care but requires rigorous validation, transparent governance, and structured clinician training to ensure safe, equitable, and ethically aligned deployment. Responsible, human-centered integration – anchored in oversight, algorithmic stewardship, and interdisciplinary collaboration – will be key to realizing full potential of artificial intelligence in trauma medicine.

**KEY POINTS**

- Artificial intelligence is increasingly applied across the trauma continuum, enhancing triage accuracy, imaging interpretation, and clinical decision-making.
- Most artificial intelligence tools remain in early validation stages, requiring robust external testing before widespread clinical use.
- Ethical governance, transparency, and human oversight are essential to ensure safe and accountable artificial intelligence deployment in trauma care.
- The WHO ethical framework and the European Union Artificial Intelligence Act define key principles for trustworthy and equitable implementation.
- Structured clinician training and algorithmic stewardship are crucial to support responsible, human-centered artificial intelligence integration in emergency and critical care settings.

## INTRODUCTION

**FIGURE 1.**
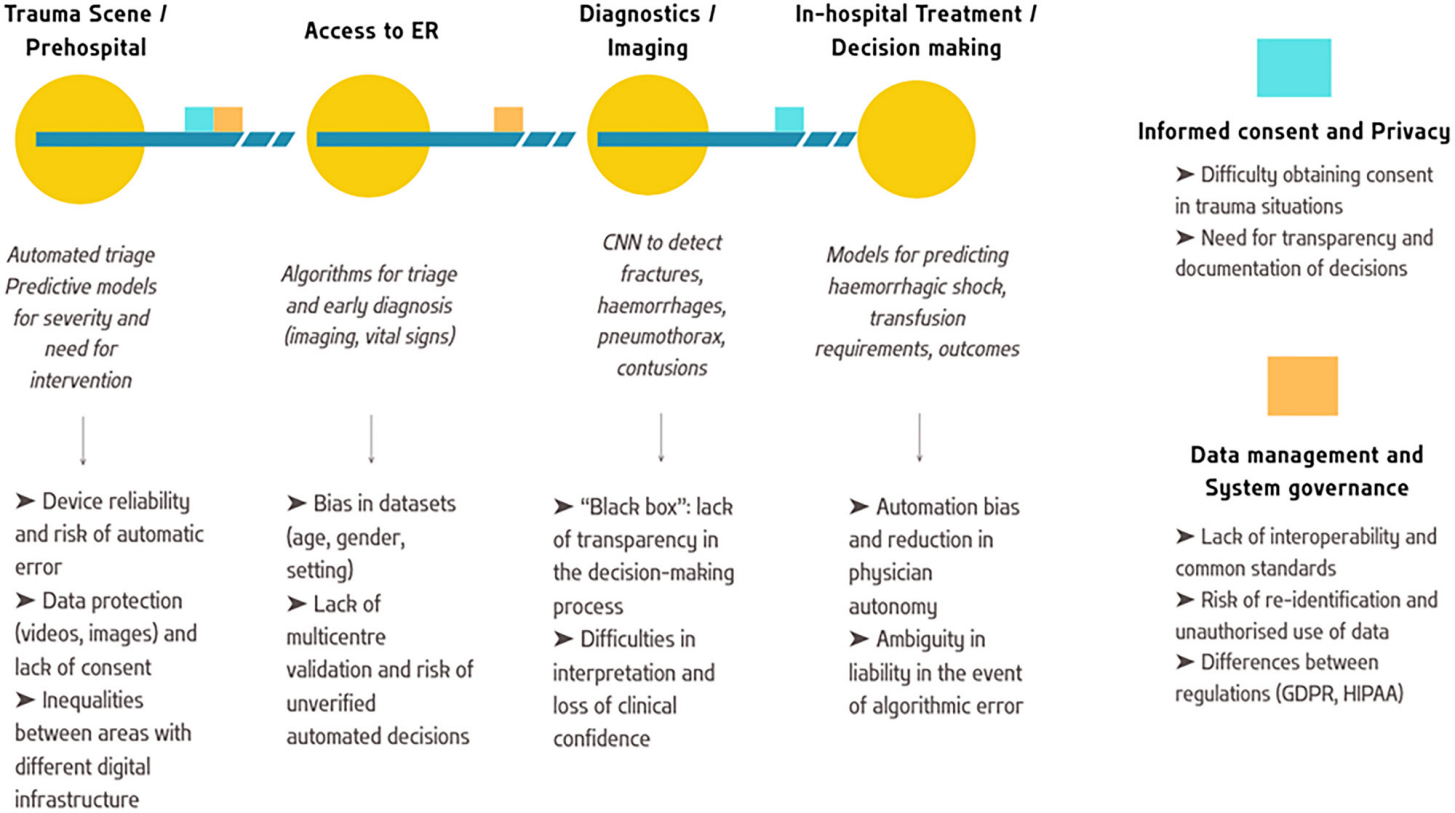
Ethical checkpoints along the trauma patient journey. The figure illustrates the progressive integration of AI along the trauma pathway – from prehospital triage to in-hospital decision-making – and highlights key ethical challenges arising at each stage. AI, artificial intelligence; ER, emergency room; GDPR, General Data Protection Regulation, HIPAA, Health Insurance Portability and Accountability Act.

The application of artificial intelligence to trauma care represents one of the most dynamic and rapidly evolving frontiers of clinical innovation. The integration of machine learning and deep learning algorithms into prehospital, diagnostic, and intra-hospital workflows aims to improve triage accuracy, diagnostic speed, and prognostic prediction. In this context, artificial intelligence, understood as the set of models and algorithms capable of learning from data and supporting clinical decisions, represents a potential tool for transforming trauma care, from prehospital intervention to the first 24 h of hospital treatment (Fig. 1).

### Artificial intelligence applications in prehospital and early in-hospital trauma care

In prehospital trauma triage, machine learning algorithms have been used to rapidly classify patients according to injury severity. Tahernejad *et al*. [1^■^] demonstrated that deep learning models, such as YOLO and OpenPose, can analyze field images or videos to estimate the number of injured individuals and prioritize interventions. These systems improve speed and consistency but raise challenges concerning device reliability and data protection. More advanced systems integrate artificial intelligence with drones equipped with thermal cameras and 5G connections, enabling remote triage in inaccessible areas. Weidman *et al*. [2^■■^] expanded this concept, using continuous waveform data from 2809 trauma patients to train an ensemble model predicting the need for life-saving interventions up to 15 min in advance, suggesting its potential for real-time decision support during field transport. Chenais *et al*. further note that, despite these advances, most artificial intelligence tools in emergency and trauma care remain in the proof-of-concept phase, with limited validation and uncertain impact on patient outcomes. They emphasize the need for independent model verification – through validation, verification, and impact assessment – before widespread clinical adoption [3^■^].

### Diagnostic and predictive applications

Artificial intelligence has become a central tool in trauma diagnostics, especially in radiological imaging. Convolutional neural networks (CNNs) have proven highly effective for automated recognition of fractures, hemorrhages, and contusions. Zhao *et al*. [4] demonstrated that, in blunt chest trauma, CNNs can detect rib fractures and pneumothorax with accuracy comparable to expert radiologists, while also quantifying lung contusions.

Similarly, Hibi *et al*. [5] found that artificial intelligence models can classify intracranial hemorrhages, estimate blood volume, and detect signs of intracranial hypertension, reducing inter-observer variability. In musculoskeletal trauma, other studies described models capable of classifying complex fractures and predicting complications, though most remain limited to retrospective data, emphasizing the need for multicenter validation [6,7]. In emergency imaging, Levy *et al*. evaluated CNNs for automatic interpretation of Focused Assessment with Sonography in Trauma exams. Using 6650 de-identified ultrasound images from 109 trauma cases, the DenseNet121 model achieved 94% accuracy for detecting free peritoneal fluid and 97% for assessing exam adequacy.

The system effectively standardized diagnostic quality across operators and, according to the authors, could enhance decision-making and training, particularly in resource-limited or high-acuity trauma settings [8].

### Prediction of hemorrhage and transfusion needs

Early prediction of hemorrhage and transfusion needs is critical in trauma care. Rapid identification of patients at risk of massive hemorrhage enables timely resuscitation, optimal blood product management, and targeted surgical intervention, ultimately improving survival outcomes. Artificial intelligence tools have been developed to predict hemorrhage and transfusion requirements. Oakley *et al*. [9^■^] reviewed 25 machine learning models, showing that those based on neural networks, Bayesian regression, and decision trees achieved area under the receiver operating characteristic curve values above 0.9, outperforming traditional scores like the ABC and Shock Index, though most studies lacked external validation. Convertino *et al*. [10] confirmed the value of the compensatory reserve index, derived from arterial waveform analysis, in detecting hemodynamic compromise earlier than vital signs. These systems could improve early recognition of hemorrhagic shock but require prospective validation and ethical governance for safe integration.

## ETHICS AND PRIVACY

Despite the rapid expansion of artificial intelligence applications in trauma care, most systems remain constrained by limited real-world applicability, lack of external validation, and uncertain clinical impact. These limitations underscore the need for an ethical and regulatory framework capable of ensuring safety, transparency, and equitable integration (Table 1).

**Table 1.** Ethical challenges and technical countermeasures in artificial intelligence-assisted trauma care

Application	Reference	Ethical issues/critical points	Practical and technical solutions
Prediction of blood loss and transfusion requirements	Oakley *et al*. [9^■^]; Convertino *et al*. [10]	Retrospectivity/the absence of external calibration: risk of premature/automated use without verification	External/prospective validation; human oversight
Management of trauma systems and governance	Stonko *et al*. [11]	The absence of a clear regulatory framework and public oversight; lack of transparency and traceability of models; difficulty in integrating data between centers	Creation of coordinated regulatory frameworks; interoperability between trauma registries; periodic re-evaluations and audits of models; national coordinating bodies for monitoring and standardization
Protection of privacy and sensitive data	Liu *et al*. [12]; Chenais *et al*. [3^■^]; Khalid *et al*. [13^■^]	Vulnerability of health data; risk of patient re-identification; legal constraints on data sharing	Synthetic data generation; differential privacy; federated learning; homomorphic encryption; blockchain
Informed consent in trauma and AI	Iserson [14^■^]; Rose and Shapiro [15]	Impossibility of obtaining explicit consent in an emergency; conflict between privacy and clinical necessity; lack of standardized criteria for the use of AI without consent	Application of the principles of necessity and proportionality to justify the use of AI; differentiated notification or consent based on the autonomy and risk of the system; transparency and documentation of decisions in cases where consent is not possible
Clinical autonomy and automation bias	Gauss *et al*. [16]; Montomoli *et al*. [17^■■^]	Risk of decision-making dependence on the algorithm; reduction in clinical autonomy; loss of critical judgement; lack of structured human supervision	Human-in-the-loop approach; algorithmic stewardship framework; periodic audits; transparency and traceability; definition of margins of uncertainty to promote medical control and professional responsibility
Transparency and explainability of models	Laur and Wang [6]; Montomoli *et al*. [17^■■^]	Lack of transparency in deep learning models (‘black box’); difficulty for clinicians in understanding and verifying decision-making processes; risk of reduced trust and professional accountability	Adoption of XAI approaches, including saliency mapping and decision path visualization; periodic audits and model traceability; documentation of updates; algorithmic stewardship to ensure transparency and accountability throughout the AI lifecycle
Training and professional culture	Chenais *et al*. [3^■^]; Boonstra *et al*. [18]; Bignami [19]; WHO [20]	Staff unfamiliarity with AI principles; fear of professional replacement; need for documented expertise for high-risk systems	AI literacy programmes; multidisciplinary training
Governance and human oversight	WHO [20]; AI Act (2024)	Risk of loss of human control and lack of traceability in algorithmic decisions; need for verifiability and clinical accountability	Mandatory human supervision; full traceability of decisions; periodic audits and monitoring; post market monitoring
Accountability and legal responsibility	Gauss *et al*. [16]	Ambiguity in defining clinical and legal responsibility in the event of algorithmic error; risk of ‘distributed responsibility’ between doctor, institution, and developer	Implementation of clinician-in-the-loop models; traceability and transparency of decisions; development of regulatory frameworks that clarify the boundaries of accountability; independent validation and continuous auditing of AI systems

AI, artificial intelligence; XAI, explainable AI.

### Governance and human oversight

The WHO guidance on ethics and governance of artificial intelligence for health defines autonomy, safety, transparency, fairness, and accountability as core principles [20]. These values, which apply not only to trauma care but to all areas of medicine, require that artificial intelligence systems support rather than replace clinical judgment. Human oversight thus becomes the balance point between computational efficiency and professional responsibility. The European Union Artificial Intelligence Act (Regulation 2024/1689) reinforces these principles by mandating continuous postmarket monitoring, traceability of decisions, and periodic audits for all high-risk medical systems. It requires comprehensive technical documentation and operational logging to allow ex post verification of algorithmic outputs. Comprehensive documentation and logging ensure ex post verification of algorithmic performance and human control, making accountability operationally traceable. Alongside, the Artificial Intelligence Act classifies these as high-risk systems, mandating effective human oversight and clearly documented accountability procedures within healthcare institutions [21].

### Global frameworks and data protection challenges

Data protection remains one of the most pressing ethical concerns in the clinical adoption of artificial intelligence. Khalid *et al*. highlight that privacy frameworks differ significantly across jurisdictions. Within the European Union, the General Data Protection Regulation governs all personal data processing and international data transfers, whereas in the USA, the Health Insurance Portability and Accountability Act (HIPAA) establishes standards for health information privacy and security. However, HIPAA (enacted in 1996) was not designed for the realities of contemporary digital ecosystems and often fails to address risks related to large datasets, cloud-based artificial intelligence platforms, and generative models. Consequently, persistent vulnerabilities remain, including risks of re-identification, unauthorized data use, and exploitation of electronic health records.

To mitigate these issues, Khalid *et al*. [13^■^] propose the adoption of privacy-preserving artificial intelligence techniques such as federated learning, differential privacy, homomorphic encryption, and blockchain, which enable secure, decentralized data processing while maintaining model performance and ethical transparency. Similarly, Chenais *et al*. [3^■^] note that real-time data use in emergency environments introduces additional challenges of compliance and oversight. Federated learning offers a practical response by allowing multi-institutional model training without transferring raw patient data, thus reducing the risk of re-identification. A complementary solution, described by Liu *et al*., involves the use of synthetic data generation through generative adversarial networks and variational autoencoders, which can replicate the statistical structure of real datasets without revealing personal identifiers. Combined with differential privacy and federated architectures, these approaches represent effective strategies for privacy-preserving research and ethical algorithm validation [12].

In line with these findings, the American Academy of Otolaryngology report by Ayoub *et al*. underscores that HIPAA is outdated and insufficient to regulate modern artificial intelligence applications. The authors call for urgent modernization of US privacy law and caution clinicians against uploading identifiable data to noncompliant artificial intelligence systems [22]. Likewise, Stonko *et al*. examine governance challenges in large-scale trauma systems, identifying the lack of coherent regulatory frameworks and limited institutional oversight as barriers to safe and coordinated artificial intelligence implementation. They emphasize the need for interoperability between trauma registries, periodic model re-evaluation, and independent auditing, alongside the establishment of national or regional coordinating bodies to define standards and ensure transparency across trauma networks [11] (Fig. 1).

### Informed consent

In trauma and emergency medicine, obtaining informed consent for the use of artificial intelligence poses a significant ethical and practical challenge. Iserson emphasize that in emergency medicine, patient autonomy and informed consent are central ethical duties, yet often difficult to uphold when patients are unconscious or critically unstable. In these cases, the author suggests that data processing and artificial intelligence-assisted decision-making must follow existing legal exceptions for emergency care, ensuring that privacy protection does not impede life-saving interventions [14^■^]. Building on this, Rose and Shapiro propose a detailed ethical framework to guide consent and disclosure in artificial intelligence-assisted healthcare [15]. The framework differentiates between artificial intelligence applications that require full informed consent – such as autonomous or diagnostic systems that significantly alter clinical standards – and those where patient notification suffices, including supportive or ‘clinician-in-the-loop’ tools. The decision depends on five criteria: the system’s level of autonomy, deviation from standard practice, degree of patient interaction, clinical risk, and administrative burden. Applied to trauma care, this model provides a practical hierarchy for ethically implementing artificial intelligence under time-critical conditions, promoting transparency, patient trust, and accountability even in the absence of conventional consent (Fig. 1).

### Transparency and explainability of models

Alongside data representativeness, transparency and interpretability remain fundamental prerequisites for trustworthy artificial intelligence in clinical practice. Data representativeness refers to the extent to which training datasets accurately reflect the diversity of real-world patient populations and clinical contexts, ensuring fairness and generalizability. Transparency denotes the clarity and openness with which an artificial intelligence system’s design, data sources, and decision-making processes are documented and communicated. Interpretability, closely related but distinct, concerns the ability of clinicians to understand how and why an algorithm produces a given output, enabling critical evaluation and responsible clinical use. The WHO identifies explainability as an essential ethical principle, recognizing that although full interpretability is not always achievable, particularly for complex deep learning models, it is crucial for accountability in high-risk applications such as trauma care [20]. Laur and Wang emphasize that many CNN used in musculoskeletal imaging operate as ‘black box’ systems, where the decision-making process cannot be directly understood. This opacity may reduce clinicians’ confidence and hinder critical evaluation of algorithmic results. The authors suggest enhancing explainability through techniques such as saliency mapping or visualization of decision pathways, ensuring both transparency and usability [6].

Montomoli *et al*. reinforce this through algorithmic stewardship, advocating systematic audits, update documentation, and explainable artificial intelligence for traceability and clinician confidence [17^■■^].

In line with these principles, the Artificial Intelligence Act mandates transparency for all high-risk artificial intelligence systems, requiring that they be designed and documented in a way that enables users to interpret their outputs correctly and understand their capabilities, limitations, and performance parameters, thus ensuring informed and accountable clinical use [21].

### Automation bias and clinical autonomy

Gauss *et al*. highlight that the integration of artificial intelligence into trauma care introduces the ethical risk of automation bias, whereby clinicians may rely excessively on algorithmic outputs, potentially compromising independent judgment and situational awareness. To mitigate this, the authors emphasize that artificial intelligence should function strictly as a decision-support tool, maintaining clear boundaries between human expertise and automated analysis [16].

Montomoli *et al*. further expand this perspective through the concepts of algorithmic stewardship and algor-ethics, frameworks designed to preserve human autonomy and accountability throughout the artificial intelligence lifecycle. They advocate for continuous auditing, explainable interfaces, and structured human-in-the-loop supervision, ensuring that clinicians remain central to decision-making and ethically responsible for the ultimate course of care [17^■■^].

### Accountability and legal responsibility

In the same article from 2024, unresolved accountability questions when artificial intelligence contributes to clinical errors are highlighted [16]. When automated triage or diagnostic systems contribute to clinical error, current regulations provide no clear definition of who is ultimately responsible – the clinician, the healthcare institution, or the software developer. This uncertainty, described as a form of distributed responsibility, represents a major ethical and legal concern in trauma settings where decisions are time critical. The authors advocate for maintaining clinician-in-the-loop models, ensuring that medical professionals retain final decision-making authority. They further recommend transparency, traceability, and independent validation of artificial intelligence tools, as well as the development of shared regulatory frameworks to clarify accountability boundaries and support safe, legally sound integration of artificial intelligence into trauma care [16].

## POPULATION AND BIAS

The reliability and clinical validity of artificial intelligence systems depend primarily on the quality and representativeness of the data used for model training. In traumatology, where patient populations are highly heterogeneous in terms of age, sex, mechanism of injury, and care setting, the risk of introducing bias – systematic distortions that affect algorithmic outcomes – is particularly relevant.

Bias in trauma-related datasets primarily stems from three factors: population representativeness, variability of clinical contexts, and data quality. Differences between adult and pediatric patients, trauma mechanisms, and institutional volumes often lead to datasets that lack generalizability. Misir *et al*. [7] report that most artificial intelligence studies in orthopedic traumatology rely on small, retrospective datasets with limited external validation, restricting broader applicability. Similarly, Laur and Wang note that deep learning models for musculoskeletal imaging are often developed in specialized centers using nonstandardized protocols and rarely tested on independent populations. Consequently, models trained in specific contexts may perform inconsistently elsewhere, affecting both accuracy and clinical equity [6]. Likewise, some authors highlight a selection bias related to the predominance of data from high-volume trauma centers, which limits model generalizability and undermines performance in peripheral or less specialized institutions [11].

In this context, Mehrabi *et al*. emphasize that bias may arise not only from data but also from algorithms themselves, producing unfair or discriminatory outcomes even in apparently objective systems. They describe multiple forms of bias – including representation, measurement, and aggregation bias – and underline that well-intentioned models can perpetuate structural inequities when trained on unbalanced datasets. Their taxonomy of fairness definitions underscores the need for transparency and systematic auditing to ensure equitable artificial intelligence deployment across populations [23].

The WHO emphasizes that artificial intelligence systems must be trained and validated on diverse, representative datasets and independently evaluated across centers to ensure fairness, robustness, and safety in clinical application [20].

## CLINICAL APPLICABILITY AND TRAINING

The successful implementation of artificial intelligence in trauma care depends not only on the technological maturity of algorithms but also on their integration into clinical workflows and on the training of healthcare professionals who use them. While artificial intelligence systems show remarkable predictive capabilities, their translation into real-world trauma settings remains limited by challenges of validation, interoperability, and standardization.

### From predictive to actionable and integrated models

In trauma medicine, most current artificial intelligence models remain confined to experimental or pilot phases, offering predictions – such as transfusion requirements or mortality risk – without clear operational guidance. Several authors distinguish between predictive models, which estimate probabilities, and actionable models, which transform predictions into therapeutic strategies. The latter, grounded in counterfactual causal inference, simulate alternative interventions (e.g. anticipating surgery or modifying blood product combinations), thereby providing more clinically relevant decision support in acute settings [16].

Integrating clinical domain expertise with machine learning is therefore essential to develop systems capable of informing real-time intra-hospital decisions rather than functioning as retrospective analytical tools. As noted by Chenais *et al*., most existing artificial intelligence applications in emergency medicine are currently used for triage optimization, patient flow management, and diagnostic assistance. However, their measurable effect on patient outcomes remains unproven, as the majority of studies are retrospective and lack experimental validation. Independent verification, validation, and impact assessment are thus required before deployment [3^■^]. In alignment, WHO recommends that all healthcare artificial intelligence systems undergo external evaluation prior to implementation, ensuring patient safety, data reliability, and compliance with local clinical practices [20].

**FIGURE 2.**
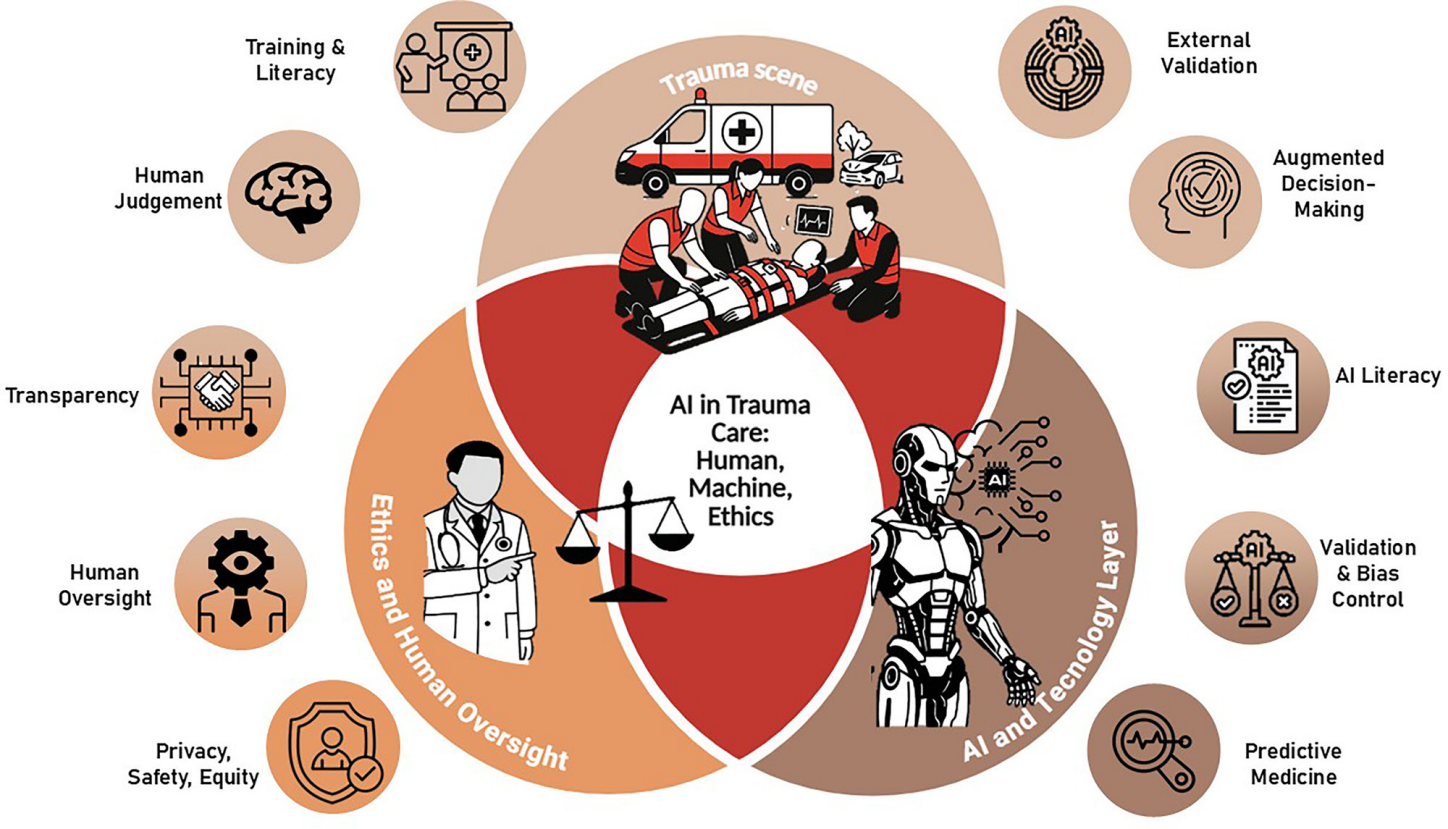
Conceptual framework of AI integration in trauma care. This Venn-inspired schematic representation illustrates the dynamic interconnection between clinical practice, AI, and ethical governance. Each domain reinforces and depends on the others: human judgment provides contextual interpretation and accountability; AI technology enhances precision, prediction, and efficiency; and ethical governance ensures transparency, privacy, and fairness. Together, these dimensions form an integrated ecosystem in which responsible, human-centered AI supports (rather than replaces) clinical judgment, enabling predictive, adaptive, and accountable decision-making across the trauma care continuum. AI, artificial intelligence.

The introduction of artificial intelligence-based tools is also transforming clinical work design, particularly in emergency and intensive care. Automation may reduce clinicians’ cognitive load but also risks altering professional responsibilities and promoting overreliance on algorithmic recommendations. Preserving the centrality of clinical judgment is therefore imperative, ensuring that artificial intelligence supports rather than supplants human decision-making [18]. In trauma care, artificial intelligence systems must remain intuitive, adaptive, and seamlessly integrated into workflows, reinforcing rather than diluting professional accountability. This balance between human judgment, technological assistance, and ethical governance is illustrated in Fig. 2, which depicts the conceptual framework of responsible artificial intelligence integration in trauma care.

### Barriers, governance, and equity in clinical implementation

Common barriers identified across the literature include poor interoperability between information systems, lack of standardized validation criteria, professional resistance, and legal uncertainty regarding liability for algorithmic errors [3^■^,^16^]. Bignami *et al*. emphasize that overcoming these challenges requires the establishment of structured governance and compliance frameworks, in line with the Artificial Intelligence Act. Their checklist-based methodology guides institutions through ethical, operational, and legal dimensions of artificial intelligence adoption, promoting transparency and fairness [19]. Consistently, both the Artificial Intelligence Act and WHO advocate for collaborative governance involving developers, clinicians, and institutional leaders, fostering shared responsibility and trust in the clinical use of artificial intelligence technologies [20,21].

In prehospital and emergency contexts, Mallon *et al*. highlight the additional ethical challenges of implementing artificial intelligence in settings with limited technological infrastructure and data governance. Algorithms trained on narrow or noninclusive datasets risk reinforcing existing inequities, particularly in low-resource environments. To mitigate these disparities, the authors recommend context-sensitive governance, early stakeholder engagement, and the use of secure, cloud-based or mobile infrastructures to ensure reliable, real-time data collection.

Progressive digitalization of emergency registries and targeted staff training are identified as prerequisites for equitable and sustainable artificial intelligence integration [24].

Finally, the global imbalance in artificial intelligence research between high-income countries and low- and middle- income countries further complicates equitable implementation. Most artificial intelligence developments and publications originate from high-income settings with greater access to computational and digital resources, risking the marginalization of low- and middle- income countries and the widening of the digital divide in healthcare innovation [25].

### Training and professional literacy

Training represents the essential bridge between innovation and clinical practice. One of the primary obstacles to artificial intelligence implementation in trauma care is clinicians’ limited understanding of algorithmic logic and reliability assessment. According to Bignami *et al*. [19], the Artificial Intelligence Act mandates structured artificial intelligence literacy programs for healthcare staff, comprising baseline education, specialized training modules, and continuous professional development incorporating legal, ethical, and governance aspects, with competencies formally documented. This approach aligns with WHO recommendations, which advocate for continuous education to enhance digital competence and critical appraisal, making artificial intelligence literacy both an ethical obligation and a practical prerequisite for the safe and effective integration of artificial intelligence into clinical environments [20].

## CONCLUSION

Artificial intelligence offers transformative potential in trauma care, improving triage, diagnostics, and decision-making. However, the current literature addresses ethical and regulatory challenges only partially. Most studies prioritize algorithmic accuracy, while aspects such as consent, fairness, and accountability remain underexplored. Evidence shows progress in technical capability but limited in systemic implementation and governance. Frameworks such as the WHO guidelines, Artificial Intelligence Act, and algorithmic stewardship provide ethical direction, yet widespread application is inconsistent.

Overall, the literature acknowledges challenges but rarely resolves them comprehensively. Future efforts must focus on multidisciplinary collaboration, external validation, and continuous ethical auditing to ensure that artificial intelligence augments, rather than replaces, human judgment in trauma medicine.

## Acknowledgements

*The authors would like to thank Dr Francesco Marconi for his valuable contribution to the preparation of this manuscript.*

*All authors contributed to the study conception and design, and read and approved the final manuscript. Material preparation, data collection and analysis were performed by E.B., M.B., and V.B. The first draft of the manuscript was written by E.B., V.B., and M.B., and all authors commented on previous versions of the manuscript.*

## Financial support and sponsorship

*None.*

### Conflicts of interest

*The authors have no conflicts of interest.*
